# A Study of the Relationship between Weather Variables and Electric Power Demand inside a Smart Grid/Smart World Framework

**DOI:** 10.3390/s120911571

**Published:** 2012-08-27

**Authors:** Luis Hernández, Carlos Baladrón, Javier M. Aguiar, Lorena Calavia, Belén Carro, Antonio Sánchez-Esguevillas, Diane J. Cook, David Chinarro, Jorge Gómez

**Affiliations:** 1 CIEMAT (CEDER), Autovía de Navarra A15, salida 56, 42290 Lubia (Soria), Spain; 2 Dpto. TSyCeIT, ETSIT, Universidad de Valladolid, Paseo de Belén 15, 47011 Valladolid, Spain; E-Mails: cbalzor@ribera.tel.uva.es (C.B.); javagu@tel.uva.es (J.M.A.); lcaldom@ribera.tel.uva.es (L.C.); belcar@tel.uva.es (B.C.); antsan@tel.uva.es (A.S.-E.); 3 School of Electrical Engineering and Computer Science, Washington State University, Pullman, WA 99164, USA; E-Mail: cook@eecs.wsu.edu; 4 Escuela de Ingeniería Informática, Universidad de San Jorge, Campus del Parque Tecnológico Walqa, 22197 Cuarte, (Huesca), Spain; E-Mail: dchinarro@usj.es; 5 Dpto. Sistemas Informáticos y Programación, Facultad de Informática, Universidad Complutense de Madrid, Juan del Rosal 8, 28040 Madrid, Spain; E-Mail: jjgomez@fdi.ucm.es

**Keywords:** Smart Grid, microgrid, Smart City, Smart Environment, Smart World, weather variables, electric power demand, sensor

## Abstract

One of the main challenges of today's society is the need to fulfill at the same time the two sides of the dichotomy between the growing energy demand and the need to look after the environment. *Smart Grids* are one of the answers: intelligent energy grids which retrieve data about the environment through extensive sensor networks and react accordingly to optimize resource consumption. In order to do this, the *Smart Grids* need to understand the existing relationship between energy demand and a set of relevant climatic variables. All smart “systems” (buildings, cities, homes, consumers, *etc.*) have the potential to employ their intelligence for self-adaptation to climate conditions. After introducing the *Smart World*, a global framework for the collaboration of these smart systems, this paper presents the relationship found at experimental level between a range of relevant weather variables and electric power demand patterns, presenting a case study using an agent-based system, and emphasizing the need to consider this relationship in certain *Smart World* (and specifically *Smart Grid* and *microgrid*) applications.

## 1. Introduction

We are living a time of change marked by governments' attempts to promote energy efficiency and renewable energy sources against an increasing power demand. In the last years, conventional power supply models are being replaced with new ones capable of meeting these two challenges at the same time. One strategy is to understand the existing relationship between energy demand and climatic variables, so as to better forecast and adapt the former to the later *in real time*. In order to do drive this adaptation, the *Smart Grids* count with huge sensor networks to exhaustively measure all kinds of power generation, power demand and climate variables in real time.

Social and political structures are increasingly concerned about climatic change and environmental awareness. In [1], Pili-Sihvola *et al.* projected the gradual impact of global warming and climate change using a multivariate regression model for five European countries from north to south. In [2], Messaoud and Chen assess how climate change affects the height growth of different tree species in the region of British Columbia, showing that variations in weather variables affect the height growth of a range of plants. Parkpoom and Harrison [3] in their work presented how climate change will affect electric power demand in the long term in Thailand by using regression models to obtain the correlations between power demand patterns and temperature. Among the many factors influencing energy demand, a number of studies have demonstrated that weather variables influence energy consumption patterns. Engle *et al.* [4] in their work present how a number of semi-parametric estimations are made on the relationship between climate and energy demand. Considine [5] assessed the impact of climate change on energy demand and carbon dioxide emissions. Hor *et al.* [6] presented the impact of weather variables on electric power demand in Wales and England on a monthly basis, by using regression models. Hyndman and Fan [7] used a semi-parametric additive model to estimate the relationship between electric power demand, temperature, working days and demographic and social factors, to predict peak loads in the long term. As the relationship between electric power demand and temperature is not linear, another study empirically investigates this non-linearity, using both parametric and non-parametric methods, as shown by Henley and Peirson [8]. Demand is driven by differences between outdoor and indoor temperature, when such difference is significant heating/cooling demand rises. The analysis performed by Terasvirta and Anderson in [9] proposes a set of smooth-transition autoregressive models for the evolution from a cold threshold temperature to a warm threshold temperature. As geographical factors are also essential in weather forecasting, Psiloglou *et al.* performed [10] a comparative study on electric power consumption in Athens and London.

All these studies assess how environmental and weather conditions affect the behavior of living beings. Electric power is indispensable and strategic to national economies. Consequently, electric power supply companies try to adapt power supply to the demand. The following studies present electric load forecasting models based on *Artificial Neural Network* (*ANN*), which includes weather variables. A study conducted in Korea presented a forecasting model where energy demand was predicted for specific daily hours basing on a combination of load data and temperature, and using a *Multi-Layer Perceptron* (*MLP*), according to Kim *et al.* [11]. Alfuhaid *et al.* [12] showed a *MLP*-based model that yields a load curve for the next day using the load curve, temperature and humidity of the former day in Kuwait. Senjyu *et al.* [13] propose a processing approach for 24 h ahead forecasting using temperature and load values for this specific time of the day from the previous days; subsequently a correction is performed to special days, and fuzzy logic is applied, to obtain a more precise forecast for Okinawa (Japan).

The conclusions drawn in all these studies suggest that environmental indicators and weather variables should be monitored, as they might be used as input in a set of specific applications—as it is the case of electric load forecasting, and they can represent a threat to the future of plants, animals and even humans. Furthermore, modern ICT and sensors should be exploited in electric power demand forecasting to facilitate the operation and control of forecasting systems. However, while the literature presents several studies of the relationship between weather variables and electric load, they are usually focused on large areas and regions and are not directly portable to smaller environments like *Smart Grids*. One of the advantages of these smart systems is that they are capable of providing precise answers to local problems thanks to distributed intelligence, and as such, the objective of this work is to particularize the correlation analysis to the *Smart Grid* scale using an adequate data set, and present a *Smart Grid* design to take advantage of this data in real time.

In the last years, a range of new concepts aimed at enhancing existing forecasting models has emerged: *Smart Grid* (*SG*), *Smart City* (*SC*), *etc.* Papers [14,15] affirm that *Smart Grids* will facilitate the operation and integration of renewable energy sources into the grid, as well as the storage of the electric power generated by these sources. In addition, these studies predict that *Smart Grids* will promote the regularization of the use of renewable energy sources in national and international markets. The studies referenced in [16,17] define the ubiquitous sensor network architecture used in *Smart Grids*. The studies referred to in [18,19] propose the use of input from *Smart Cities* to create services on demand and perform real-time monitoring and control. In [20–23] it is explained how to promote the integration and management of *Smart City* sensors for monitoring public spaces; in addition, these studies explain how to ensure the profitability and operation of smart applications by utilities.

Gann *et al.* [24] affirmed that cities become “*smarter*” when they exploit the increasing data and analytical techniques available, in order to improve energy effectiveness and efficiency through the integration of physical infrastructures and digital technologies. Naphade *et al.* [25] proposed that for the successful spread of smart cities, interoperability between sensors and smart devices must be achieved, and security and privacy must be guaranteed.

It is worth mentioning that extensive sensor networks are a key component of all these Smart Environments. Their intelligent behavior depend directly on the quantity, quality and completeness of the data, so all kinds of measuring devices, from smart meters to weather stations, are included in large numbers in the system.

Smart models that integrate sensors and distributed artificial intelligence offer a wide range of possibilities for the operation, automation and control of a range of systems. This would allow new applications to receive input from different models, and to assess how climate and environmental parameters impact human habits and affect the environment. Section 2 begins with a description of the conventional electric power supply system, to continue with an introduction to the new models emerged in the field of electric power supply system and other related spaces and its collaboration inside a *Smart World*. Section 3 introduces the data set employed and the methodology used for its analysis. Section 4 details the results. Section 5 describes how weather data for a specific area can be used inside a *Smart Grid* with a Multi-Agent System model. Finally, Section 6 presents the conclusions of this work and anticipates future studies.

## 2. The Conventional Electric Power Supply System. Future Approach to General Service Systems and the New Models Associated to Them

### 2.1. Conventional Electric Power Supply System

**Figure 1.**
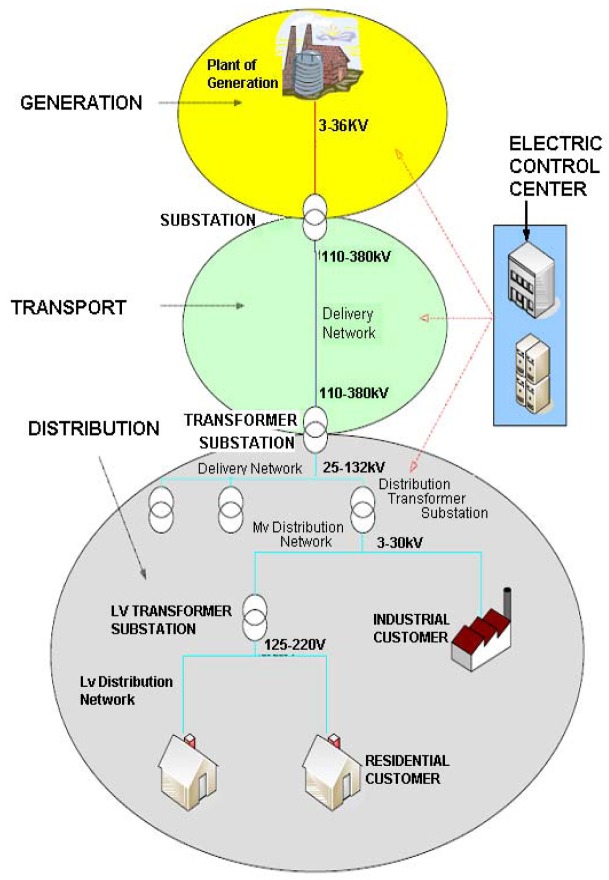
Electric Power Supply System.

A typical electric power supply system comprises three elements: plants of generation that produce electric power, transmission lines that transport electric power over long distances, and distribution lines that deliver electric power to end-users. Between these elements, there are transformer substations and transformation centers that adapt voltage to specific levels. All components are shown in Figure 1.

### 2.2. Future Power Supply Models

The conventional model presented in section 2.1 has evolved thus leading to the emergence of a new concept: the *Smart Grid*, which can be defined as a “*smart*” electric power distribution network that exploits ICT to optimize electric power generation and distribution systems. *Smart Grids* allow a more efficient adjustment of power distribution to energy demand. Simultaneously, a range of new concepts associated with *Smart Grids* has also emerged: *Distributed Energy Resource* (*DER*), *Distributed Generation* (DG), *Demand Response* (*DR*—adapting energy demand in time in a controlled way, be it either for market economic interests or for the need to adjust the demand to the existing *DG* systems, *Electric Mobility* and *Electric Vehicles* (*EV*), *Smart Meter* (*SM*—an electronic measuring system that records electric energy consumption), *Smart Home/Smart House* (energy consumption is an aspect of home life that is often overlooked, and energy consumption increased at a higher rate than the population growth, as shown Björkskog *et al.* [26]) and *Smart Customer* (*SCu*—consumers aware of their consumption patterns; according to the *European Technology Platform SmartGrids 2035* [27], the concept of *SCu* is critical and strategic).

*Microgrids* emerge as an evolution of *Smart Grids*. According to the *Consortium for Electric Reliability Technology Solutions* (*CERTS*), a *microgrid* involves an “aggregation of loads and micro-power units jointly operating as a single system to provide both electric power and heat. A *microgrid* is a system that includes power units, energy storage and interconnected loads that can operate both connected to the bulk power system and in isolation from the grid in case disturbances may arise.”

*Smart Grids* lead to the emergence of *Smart Cities*, where urban performance depends not only on the city's endowment of hard infrastructure, but also, and increasingly so, on the availability and quality of knowledge communication and social infrastructure (*intellectual and social capital*). In this context, ICT and sensors are crucial to identifying and meeting social needs and environmental standards.

In this context, a more specific concept arises: *Smart Building* (*SB*), a new model of building which integrates renewable energy sources and an intelligent management system based either on economic or self-sufficiency strategies.

*Smart Environment* (*SE*) is defined as one that is able to acquire and apply knowledge about its inhabitants and their physical surroundings to improve the performance of the environment. Smart environment technologies can be used in a variety of ways to improve energy efficiency: by analyzing electric power usage to identify trends and anomalies; by identifying correlation between human behavior and electric power consumption; and by automating environments to be more energy efficient.

### 2.3. Synergies among Models—A New Umbrella Concept

**Figure 2.**
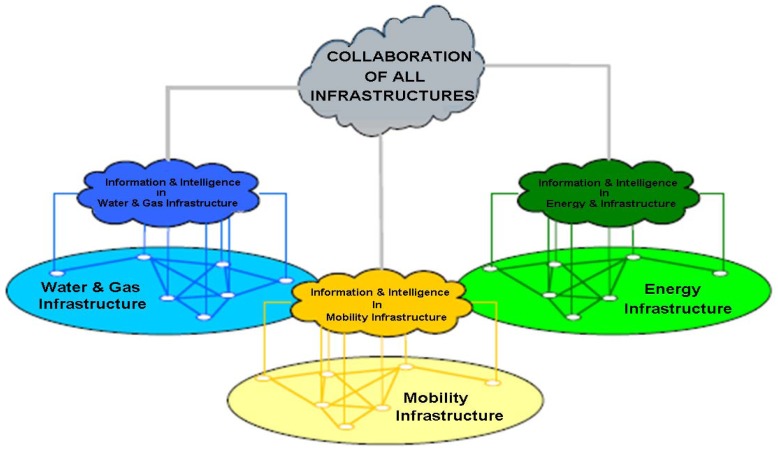
Concept of Smart Place.

Section 2.2 describes the evolution of the conventional electric power supply system into *Smart Grids* and other derived scenarios integrated in *Smart Grids. Smart Grids, microgrids, Smart Cities, Smart Buildings* and *Smart Environments* are spaces where physical infrastructures are deployed in combination with sensor networks and ICT to manage data. This situation is shown in Figure 2, where the electric power supply infrastructure might be a *Smart Grid* or a *microgrid*. Figure 2 shows a new system that would exploit the information provided by each infrastructure, to meet a set of global objectives aimed at improving energy efficiency and quality of life in cities.

As the presence of smart systems is increasing in the market, and new smart models are likely to appear in the coming years, it is important that a standardized definition of the concept “smart” is established, as smart models provide data that can be used as an input in other models for different purposes. For instance, the electric power demand from a *Smart Grid* could be associated with data from renewable energy sources controlled by the *Smart City* to improve energy efficiency in the city and demand forecasting in the grid. The *Smart World* encompasses all the interconnected communities sending and storing information from their sets of distributed services, sensor networks, infrastructures, data transmission technologies and/or control systems.

**Figure 3.**
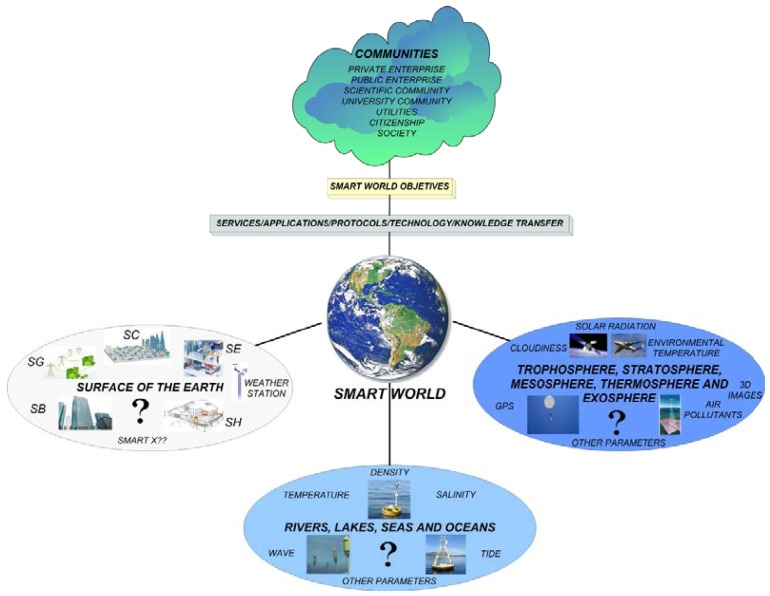
*Smart World* Concept.

*Smart World* is represented in Figure 3, where information flows from the distributed intelligence and sensors of *Smart Environments* deployed worldwide in oceans, airplanes, satellites and sounding balloons monitoring the Earth. *Smart World* will become a global scenario where different communities will exploit the information provided by a variety of data transmission technologies to achieve their specific purposes according to *Smart World's* goals.

## 3. Data Analysis, Information Processing and Methodology

This Section presents data demonstrating that a relationship exists between weather variables and electric power demand. Section 4 shows the results of data analysis. Accordingly, meteorological data should be collected from meteorological stations in order to make it available for the *Smart World*, as it is required for the development of several applications.

### 3.1. Electric Power Consumption Data—Weather Variables

**Figure 4.**
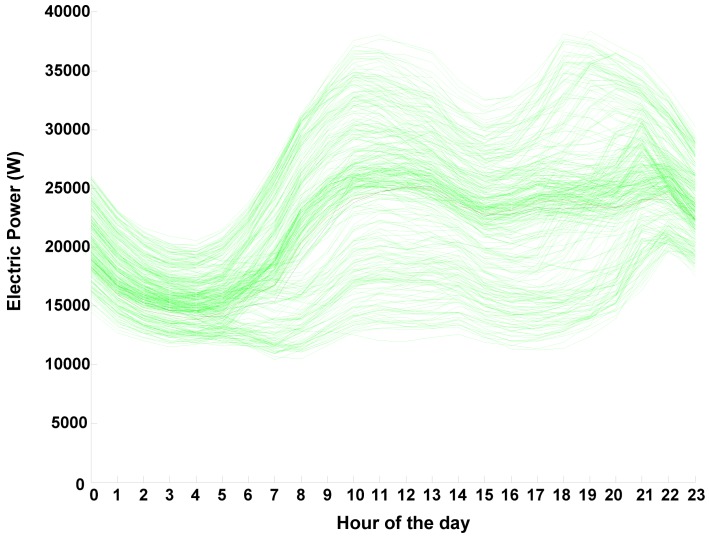
Load curves for non-holiday Wednesday are represented in green. Load curve for 30/06/2010 is represented in red, and the forecasting for that day is shown in black.

This study analyzes the electric power consumption data (electric power consumption ranged between 7 and 39 MW) provided by the Spanish electric power supply company *Iberdrola*. Data were gathered from 1 January 2008 to 31 December 2010 from a substation located in Soria. The data provided includes: day, month, year and hourly electric consumptions that form the daily load curve. The main indicator in this study is the hourly load curve, which is crucial for power suppliers and *microgrid* managers. As an example of the data employed, load curves for non-holiday Wednesdays are shown in green in Figure 4, a wide variety of load curves different in size and form can be seen in this Figure in spite of the fact that only non-holiday Wednesdays are represented. (Non-holiday Wednesdays have been chosen randomly purely for exemplification purposes; the data presents the usual variations in demand [28] depending on day of the week—with weekends showing a weaker demand-, season, *etc.*).

The meteorological data used in this study were collected by the Spanish Meteorological Agency AEMET from the meteorological station installed in Soria. The meteorological data were collected from 1 January 2008 to 31 December 2010.

The weather variables considered are: precipitation (mm), air temperature (°C), average wind speed (m/seg), average wind direction (sexagesimal degrees), relative humidity (%) and pressure (hPa). Each row includes data on: date and hour of registration, minute of registration, source meteorological station, altitude, name of the province, longitude, latitude, precipitation, ambient temperature, average wind speed, average wind direction, relative humidity, pressure and global solar radiation.

### 3.2. Information Processing

**Figure 5.**
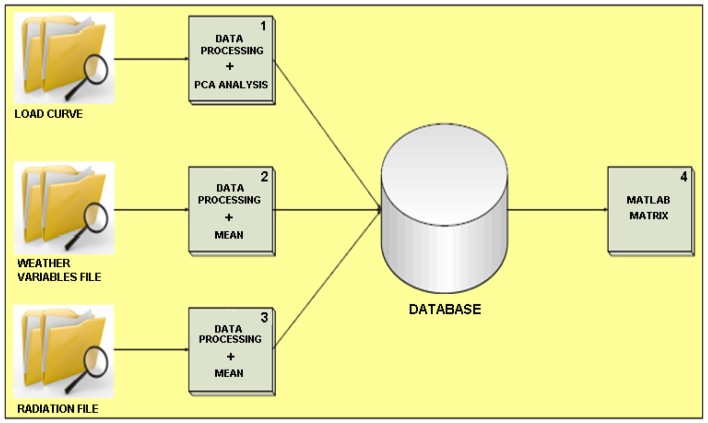
Information Processing Scheme.

Raw data are presented Section 3.1, however, once data were obtained, they were processed for the purposes of the study. Figure 5 shows the information processing scheme, which is explained below.

Processes 1, 2 and 3 were performed with raw data before their inclusion in our database. In process 4 raw data were processed and subsequently entered into MatLab. Hourly electric power consumption is displayed in load curves, which was very useful for our purposes, since—as it was mentioned above—it is a key measurement for power suppliers and grid managers. As concerns process 1, when one data was missing values were interpolated. This is done instead of discarding those values in order to implement a real time behavior that can be easily ported to a deployed network.

Another action performed afterwards is examining the load curves to perform outlier detection and discard wrong daily load curves originating from hardware failures; abnormal values which are correct, as in the case of low electric power demand in a holiday, should be distinguished from errors that might be caused by a technical failure, which are the ones that must identified and removed.

**Figure 6.**
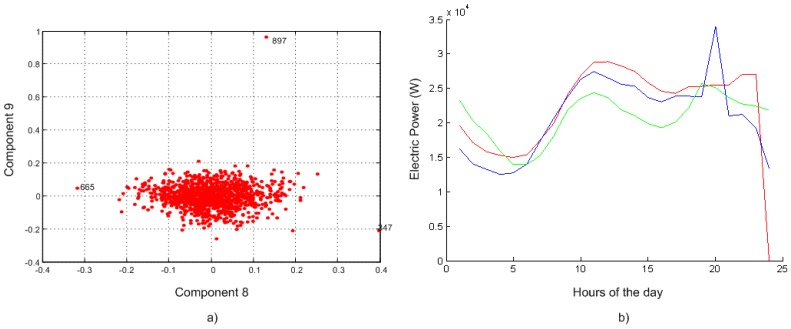
(**a**) PCA Analysis: component 8 and 9. (**b**) Blue: 665, Red: 897 and Green: 347; the number means the number of pattern in the data set.

For the detection of these outliers, the Principal Component Analysis (PCA) has been used, which is a mathematical procedure that uses an orthogonal transformation to convert a set of observations of possibly correlated variables into a set of values of uncorrelated variables called principal components. Figure 6 shows the results of PCA where (a) shows all the patterns (all data are displayed in the form of a load curve) that might be potential outliers after a comparative analysis of 8 and 9; (b) shows two outlier load curves (numbers 665 and 897 in the data set) containing wrong sensor readings as compared to a correct load curve for a special day (pattern number 347).

Weather variables—except global solar radiation—are monitored on a ten-minute basis. In process 2, the first step is to identify any missing value for a ten-minute interval and infer it using interpolation (this is done, again, to implement a system capable of working in real time). Then, air temperature, average wind speed, average wind direction, relative humidity and pressure are averaged in groups of six ten-minute intervals to obtain hourly measures. Precipitations are not averaged, but accumulated instead. Then, daily average values are calculated for all of the variables.

As the file that displays the global solar radiation provides hourly data from 5:00 AM to 20:00 PM, process 3 is exclusively performed to detect and interpolate any missing hourly datum.

Once calculations were performed, load curves—including the technical outliers detected, hourly weather variable values, and the averaged weather variable values for each day were displayed in a database. Process 4 yielded a data matrix to subsequently perform a correlation analysis using MatLab. The first step in this process is to examine all patterns (full days) excluding patterns with load curves marked as technical outliers, and patterns with too many missing values for a weather variable to be interpolated.

### 3.3. Methodology

In statistics and probability, a correlation measures the strength of the relationship between two variables. Among the several coefficients measuring this correlation degree, the one chosen for this study as the most widespread and commonly employed is the Pearson's linear correlation coefficient. It is obtained by dividing the covariance of two variables by the product of their standard deviations. Pearson's correlation can be defined as an index that measures to what extent two quantitative variables are linearly related. Correlation coefficients can range from −1.00 to +1.00:

- *r* = +1 represents a perfect positive linear correlation.
- 0.0 < |*r*| < 0.09 represents no correlation.
- 0.1 < |*r*| < 0.25 represents a small linear correlation.
- 0.26 < |*r*| < 0.40 represents a medium linear correlation.
- 0.41 < |*r*| < 1.0 represents a strong linear correlation.
- *r* = 0 indicates that both variables are not linearly related.
- −1 < *r* < 0 represents a negative linear correlation.
- *r* = −1 represents a perfect negative linear correlation.

The data matrix obtained in process 4 allows the initiation of the study. This study was aimed at determining whether a direct relationship exists between electric power demand and the set of weather variables considered.

## 4. Results

Once the MatLab experiment is performed, the solution matrix is obtained; subsequently, this matrix is analyzed to obtain the final conclusions of this study. The result of this matrix is entered into a spreadsheet for subsequent analysis. The average correlation coefficients between the different variables and the 24 consumption times are shown in Table 1.

**Figure 7.**
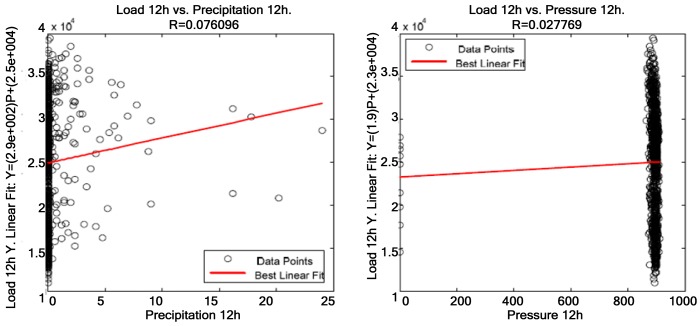
Correlation among precipitation, pressure and electric power consumption at 12 h.

**Figure 8.**
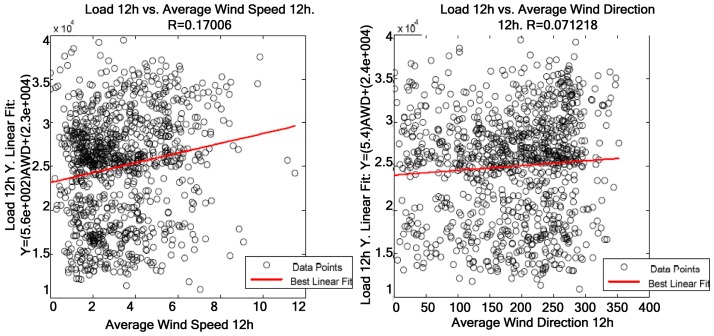
Correlation between average wind speed + average wind direction and electric power consumption at 12 h.

**Figure 9.**
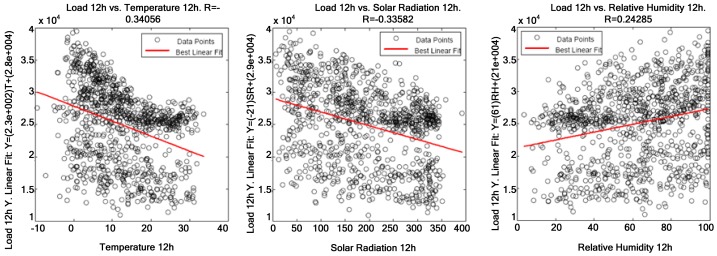
Correlation among temperature global solar radiation, relative humidity and electric power consumption at 12 h.

The table above shows that the variables most significantly correlated with electric power consumption are temperature, global solar radiation—both have a negative correlation—and relative humidity—which is positively correlated with electric power consumption. However, average wind speed and direction are not significantly correlated with electric power consumption, and precipitation and pressure are not correlated with it at all. Correlations between weather variables and electric power consumption are displayed below, having 12 h as the reference time of day. Figure 7 represents uncorrelated weather variables—precipitation and pressure—where points are widely spread around the straight line and Figure 8 displays slightly correlated weather variables. Finally, Figure 9 shows the variables that are most significantly correlated with electric power consumption.

Figures 7–9 demonstrate that a significant correlation exists among temperature, average solar radiation, relative humidity and electric power consumption. Their correlation against electric power consumption is displayed at 0 h, 5 h, 12 h, 16 h and 20 h below. The first time represents the change of day and the other four times are the closest to the four characteristic points of the load curve (two peaks and two valleys). Thus one can observe how the relationship changes as the day progresses.

**Figure 10.**
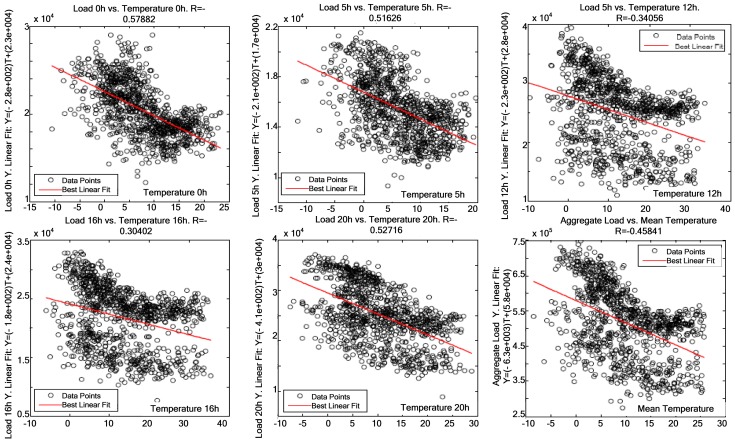
Correlation at 0 h, 5 h, 12 h, 16 h, 20 h and average temperature against electric power consumption.

Figure 10 presents the other five times of the day where measures were taken, and the average correlation between temperature and electric power consumption. The correlation coefficient decreases at 12 h and 16 h. This is due to human habits, which make consumption rise regardless of the temperature. For example, there is more activity in residential buildings at 12 h and 16 h, as electric cookers are intensely used at those times.

**Figure 11.**
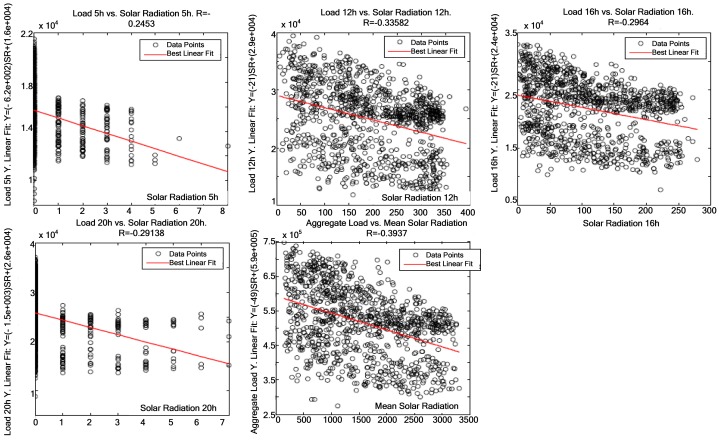
Correlation at 5 h, 12 h, 16 h, 20 h and average global solar radiation against electric power consumption.

Figure 11 presents the five most relevant times of day—except the day change (0 h) as it is irrelevant to this weather variable, and the average correlation between global solar radiation and electric power consumption. A more evident correlation is found in the middle of the day—coinciding with the maximum solar radiation, thus contrasting with 5 h and 20 h, which is when solar radiation increases and decreases respectively.

**Figure 12.**
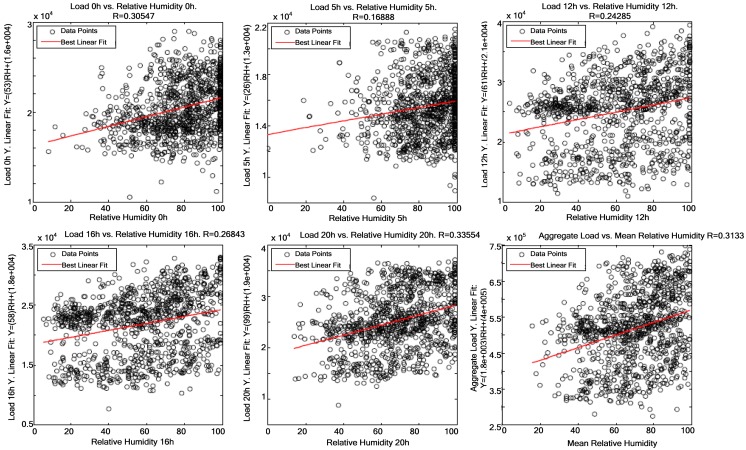
Correlations between average relative humidity and electric power consumption at 0 h, 5 h, 12 h, 16 h, and 20 h.

Figure 12 shows the five times of day where measurements were taken and the overall average correlation between relative humidity and electric power consumption. At 0 h, 5 h and 20 h most patterns are associated with relative humidity measures >60%, while the rest of time patterns are more widely scattered. The correlation is lower at 5 h, which is probably due to the fact that industrial processes are initiated at that time and have a higher influence on electric power consumption than relative humidity.

Up to now, the results have shown the average yearly correlation of the aggregate load against the environmental variables considered. While these variables show a strong seasonal behavior, the aim of this work is to study the aggregated behavior of electric load along time. This is because classifying data (and the system's behavior) according to seasons is just one of the many possible classifications, and it will be the task of the system's intelligence in a later stage (using classification and pattern recognition algorithms) to group and process the data in the most suitable way, which might or might not have a straightforward human meaning as it is the case with seasons. However, as a reference Table 2 presents the results of the correlation coefficient considering the different seasons and the most important variables.

It is easy to see the season influence in the correlation coefficient. For instance, the correlation for average temperature is high for spring and autumn, but not for Summer and Winter. This can be easily explained because in winter and summer, the temperature is always perceived by humans as “cold” and “hot” respectively, and as such, the behavior related to temperature is normally fixed under those conditions, showing a bigger dependence with other parameters.

## 5. Future Applications of Weather Variables

As the *Smart World* concept spreads worldwide, new social, political and research needs will arise and, with them, new applications requiring the interaction of different models. Section 5.1 presents some environments—*Smart City* or *microgrid*—and applications that will require weather variable measurements. In Section 5.2, a case study is presented where the relationship between climatology and power demand is examined using the Multi-Agent System (MAS).

### 5.1. Some Applications Requiring the Measurement of Weather Variables

Section 3 demonstrates that a relationship exists between some weather variables and electric power demand in a particular area—of a scale which might be considered that of a *microgrid*. As regards electric power demand, the correlation between variables from different models should be understood, as weather variables are required by *ANN*-based electric load forecasting models. In addition to using weather variables as inputs in electric load forecasting, it would be interesting to assess how the relationship between weather variables and electric power demand changes; this would allow the identification of the new variables required by new forecasting models. Moreover, the users of a *microgrid* might be informed on the changes in the relationship between weather variables and electric power demand so that they understand how weather influences their consumption habits.

For example, cities are expected to become sustainable, environmentally-friendly and make a systematic use of distributed renewable energy in the future. *Smart Cities* will have historical data available almost in real-time, which will serve to assess environmental improvements through control ratios; control ratios will serve to assess how the electric power supplied by renewable energy sources increases, as compared with conventional energy sources. Other control rations might reveal the amount of pollutants that have not been emitted thanks to the use of renewable energy sources in the *Smart City*.

In *Smart Cities*, weather variables can be also used in urban planning, as it requires an accurate forecasting of green energy generation in buildings, public spaces, *etc.* For this purpose, information on solar radiation from satellite observations, LIDAR-based 3D mapping, and the assessment of the status of power supply facilities in a particular site may help urban planners decide whether mini-wind and photovoltaic parks can be installed in that site. Similarly, planners will be provided with the information required to decide almost in real-time whether the distribution network deployed will allow the evacuation of the generated power. These applications will encourage the use of renewable energy sources and promote sustainable planning of future cities, parks, towns and public buildings. This will result in a more rational urban growth in terms of infrastructures, thus allowing the exploitation of local power generation resources (solar energy, wind, biomass, *etc.*).

### 5.2. An Implementation Proposal

A system capable of realizing the weather dependencies pointed out in this paper is not trivial to design. There are several unknown factors, like how to interact with elements of the power grid or if there are other meaningful climate variables affecting the power consumption. Nevertheless, the study can serve as basis for an initial system's architecture proposal. In the literature on *Smart Grids*, different technologies have been applied. From them, we focus on MAS, which have been already applied to *Smart Grids* in different situations [29].

Following Farhangi [30], power grid networks have been improved continuously for an increase of control, flexibility, and efficiency. The existing *Advanced Metering Infrastructure* approach is a necessary initial step towards having world-wide *Smart Grids*. In this transition, technology capable of dealing with plug-and-play of components, with some embedded intelligence, is highly needed, and a good candidate for its implementation is MAS as well. The need of increasing the intelligence in local nodes through MAS is defended also in Massoud and Wollenberg [31]. They compare a pure SCADA approach with a MAS approach, remarking the plug-and-play capabilities provided by the MAS solution and a better use of communications. The MAS technology has the required performance and scalability for dealing with a *micro grid*, such as the work performed by Jimeno *et al.* [32], who have built a *micro grid* and associated an agent to each element. These agents were capable of negotiating the workloads, what led to a greater overall efficiency and adequateness to the demand.

Because of the abovementioned reasons, we decided to experiment with an agent oriented design and implementation. As methodology, we chose INGENIAS [33], because it is one of the few supporting implementation from a generic design. Also, INGENIAS follows a model driven approach which provides enough flexibility to obtain different implementations from the same specification. Also, INGENIAS is capable of doing Agent Based Simulation using a software-in-the-loop approach [34].

**Figure 13.**
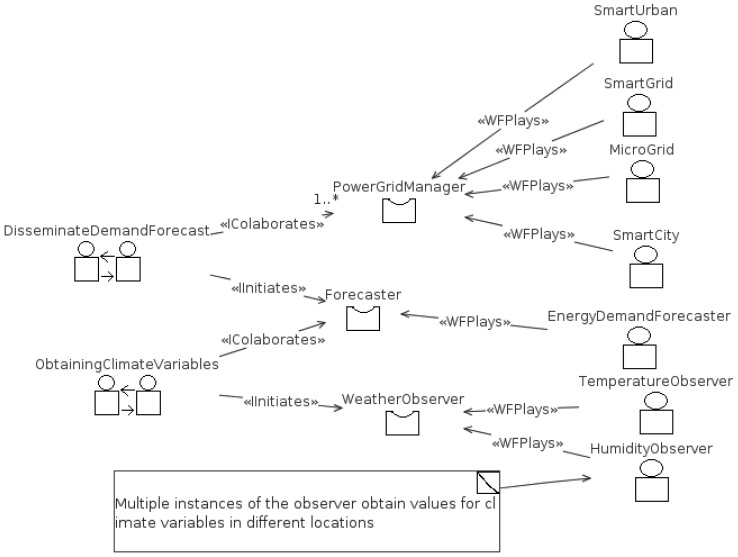
MAS design of a *Smart World* architecture taking advantage of weather data.

The result of applying INGENIAS is a MAS architecture draft for the deployment of a weather aware power grid system. Figure 13 introduces how the elements cooperate, remarking two key interactions: one to deliver weather data and another one to provide a forecast. Several sensors, represented by *Weather Observer* role, are installed worldwide to measure different weather variables. Variable values are delivered to one or several climate modeling agencies, represented as *Forecaster* role, that know how weather information is associated with the climate model they handle. They subsequently associate the evolution of climate variables with power demand. Then, the demand forecast is reported to each of the intelligent grids (*PowerGrid Manager role*) worldwide or region wide and, in exchange, they obtain feedback on the actual demand, which helps improve the climate model. Mentioned roles (*Weather Observer, PowerGrid Manager*, and *Forecaster*) are played by concrete agent types representing different kinds of power grids. It would be expected that each agent has zero or more instances, depending on the regarded scenario.

**Figure 14.**
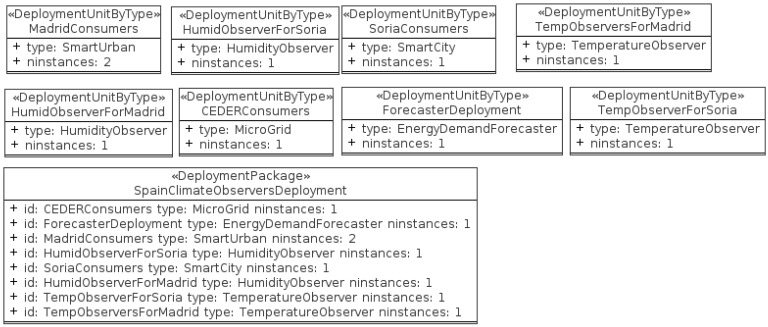
Instances of Actors in the test simulation.

In this paper, a specific deployment instantiation of that architecture has been simulated to prove that it works. It involves the definition of a set of agents which fulfill the role of the actors specified in Figure 13. This agent scheme is shown in Figure 14. It resembles the structure of some Spanish cities, specifically Madrid and Soria where real weather data are supplied to climate observer agents. Madrid and Soria provide sensors of humidity and temperature (*Temperature Observer* and *Humidity Observer* types). There are agents responsible of power management (*Smart City* type) for both cities plus another one representing a *microgrid*, the CEDER Consumers. There would be one instance of *Energy Demand Forecaster*, responsible of data analysis and forecast production.

**Figure 15.**
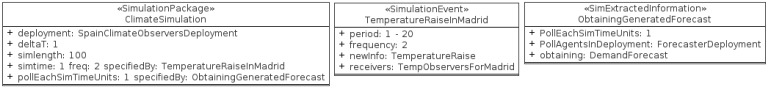
Simulation configuration for the Madrid scenario

The scenario of this simulation is presented in Figure 15. It models an agent based simulation of 100 units of simulated time. In the 20 first units, an event indicating an increase of temperature is generated each 2 time units in the *Temperature Observers* from Madrid. Each simulation time unit, the forecast is obtained for evaluation in the simulation. With this architecture, we can accommodate the algorithms producing the forecast inside the *Energy Demand Forecaster* and reproduce a close to real software deployment conditions, rather than working with raw data sets.

The resulting architecture is rather flexible and provides plug-and-play capabilities identified in [30] and [31]. Additional sensors can be deployed even in runtime and they would participate transparently in the execution. Similarly, new agents representing other actors, such as *microgrids* or *Smart Grids*, would be automatically incorporated in the notification of forecast reports. This is possible because agents make extensive use of yellow pages services each time an interaction is engaged. So, if new agents are incorporated, they must notify the agent management framework and register into the yellow pages services.

## 6. Conclusions and Future Studies

As this study presents, all smart models (*Smart City, Smart Grid, etc.*) are aimed at achieving a set of common goals: sensing the environment, monitoring the operation and performing intelligent adaptation. For this reason, this work presents the umbrella term *Smart World*, which will represent a framework for the interconnection and cooperation of several Smart Systems. The overall goals of *Smart World* could be listed as: operation, maintenance, optimization and automation of infrastructures; safety and security; mobility and transport; urban planning; energy saving and efficiency; sustainability; environmental evolution and control, and good quality of life. Regardless of talking about a *Smart Grid, microgrid, Smart City, Smart Environment, etc.*, (with their own sensors, the applications, services, protocols, *etc.*), meeting one or some of the goals mentioned above will be designed according to the general concept of *Smart World*.

Existing correlation studies between weather variables and electric load are usually focused on regions and big areas that are much bigger than *microgrids*. This work is focused on performing the analysis at *microgrid* scale, demonstrating that, even in a small scenario, a relationship still exists among a set of weather variables and electric power demand. For *Smart Grids*, systems capable of precise adaptation to particular local conditions, can greatly benefit from understanding the small-scale influence of weather in electric load. This is important because most of current *Smart Grid* deployments do not actually monitor weather variables nor employ them in their adaptation and prediction tasks. And as shown along this work, there is a lot of valuable and easy to retrieve information in those variables.

The relationship with weather variables could potentially change with time in the same way that it has changed in the past. The place where the correlation tests are performed also influences the strength of this relationship, as it will be stronger in an environment with extreme weather than in a region with stable warm weather. Therefore, as time and physical location are essential factors, applications should evolve with time, and focus on the site specific to the study, in order to obtain accurate information on the changing relationship between weather variables and electric power demand. For a proper operation, smart applications collect information from a range of systems such as the electric power supply infrastructure or the national meteorological station network with their sensors.

Therefore, some technological challenges need to be solved in order to allow a full implementation of adaptation intelligence into *Smart Grids*, opening a set of research directions. First, it is necessary to develop intelligent hardware capable of running software implementing the agents, both to complete the monitoring sensor networks and the intelligence in the *Smart Grids*. Second, most of the algorithms currently employed in power grids are designed to operate at a nation-wide scale, and it will be necessary to adapt them for operation in small environments. This is especially true (as shown along this work) for load forecasting algorithms, and there is still a lot of work to do in order to be able to predict loads at small *microgrids* and even single nodes, but it is also true that new control algorithms have to be designed to operate at a extremely small scale (with nodes being represented even by single devices). For this, the study reported along this work represents a very important step, since local weather variables will be an extremely valuable input for node load/production forecast.

Additionally, it is still necessary to design and standardize framework solutions to allow communication and interaction of the different entities in the *Smart Grid*, so as all the different industries implied (clean and small scale power production hardware, intelligent software design, home and industrial equipment, *etc.*) can focus on solving their particular challenges and forget about solving interoperability issues.

This work demonstrates that a correlation exists between weather and electric power demand, they intend to design and validate new *ANN*-based architectural models for electric load forecasting in reduced areas (corresponding to a *microgrid*), which collect information on the most relevant weather variables. Further research studies will be conducted in order to identify the new applications that will be required to meet *Smart World*'s goals. Future studies will also focus on the identification of the environments where these new applications might be implemented, assuming that these applications would collect information from different models using sensors. As in the present study, the new one will also employ the INGENIAS Development Kit (IDK), which uses a MAS-based specification language.

## Figures and Tables

**Table 1.** Correlation between weather variables and electric power consumption.

**Average Weather Variable**	**Average Correlation with 24 h**
Average precipitation	0.075
Average temperature	−0.458
Average wind speed	0.199
Average wind direction	0.186
Average humidity	0.308
Average pressure	0.0102
Average solar radiation	−0.393

**Table 2.** Seasonal correlation coefficient between weather variables and electric power consumption.

	**Spring**	**Summer**	**Autumn**	**Winter**
**Average Temperature**	−0.326	−0.077	−0.396	0.065
**Average Relative Humidity**	0.228	0.053	0.418	0.027
**Aggregated Precipitations**	0.085	0.131	0.235	0.180
**Average Pressure**	−0.138	−0.023	−0.022	−0.303
